# Pathophysiology and Symptomatology of Drooling in Parkinson’s Disease

**DOI:** 10.3390/healthcare10030516

**Published:** 2022-03-11

**Authors:** Sotirios Polychronis, Grigorios Nasios, Efthimios Dardiotis, Lambros Messinis, Gennaro Pagano

**Affiliations:** 1 Department of Speech & Language Therapy, School of Health Sciences, University of Ioannina, 451 10 Ioannina, Greece; grigoriosnasios@gmail.com; 2 Department of Neurology, University Hospital of Larissa, University of Thessaly, 412 23 Larissa, Greece; ebsdar@gmail.com; 3 Laboratory of Cognitive Neuroscience, School of Psychology, Aristotle University of Thessaloniki, 541 24 Thessaloniki, Greece; lmessinis@psy.auth.gr; 4 Exeter Medical School, White City, London W12 0HR, UK; gennaro.pagano@roche.com; 5 Neuroscience and Rare Diseases Discovery and Translational Area, Roche Pharma Research and Early Development, F. Hoffmann-La Roche Ltd., 4070 Basel, Switzerland

**Keywords:** Parkinson’s disease, salivation, clinical features, non-motor, motor, sialorrhea, drooling

## Abstract

Drooling can present in patients with Parkinson’s disease (PD), and it is manifested as an excessive pooling of saliva inside the oral cavity. Currently, the exact pathophysiological mechanism of drooling in PD is not yet fully explicated. Thus, it becomes crucial to understand if some clinical characteristics may emphasize drooling or if they are just concomitant. In PD, excessive drooling has been associated with a higher burden of non-motor symptoms, such as cognitive impairment, sleep problems, autonomic dysfunction, constipation and orthostatic hypotension, and of worse severity of motor fluctuations and bradykinesia. PD patients with excessive drooling also showed a reduction of striatal DAT availability at DaTSCAN imaging. Excessive drooling in patients with Parkinson’s cannot be attributed to a single factor but to a mixture of factors, including but not limited to impaired nigrostriatal pathways.

## 1. Introduction

Drooling is commonly manifested among patients with Parkinson’s disease, and it can be caused by the excess production of saliva, inability to retain saliva within the mouth (incontinence of saliva), or problems with swallowing (dysphagia) [1]. It can be caused due to a hyper-production of saliva inside the oral cavity or a change in salivary clearance as a result of swallowing impairments or difficulty in containing saliva inside the oral cavity [1]. Research studies have examined the pathophysiology of drooling in PD [2,3,4,5,6,7,8,9,10,11,12,13,14,15,16] in order to better understand the relationship between drooling and the clinical symptoms in PD. Over the course of the disease, the prevalence of drooling varied between 9.26% and 70% [17,18,19,20,21], it is higher in males [19,22,23,24] than in females and the longer the disease duration [17,18,24,25] and progression, the higher the risk of drooling [14,17,18,22,24,25,26]. Moreover, the higher the age and the severity of Levodopa-induced dyskinesia, the more prevalent the drooling is. A single work reported that drooling might, in some cases, be a prodromal PD symptom [20]. Even though inconsistent findings have been suggested regarding the association between the cognitive performance and drooling [17,22,23,25,27,28,29,30], it appears that drooling is related to sleeping disorders [22,23], dysautonomic symptoms [22,23,31], speech difficulties [23], dysphagia [17,23,25,32,33,34,35], hypomimia [24,26,36], bradykinesia [26], and a more symmetric pattern of PD presentation [24,37]. Neuroimaging research studies have suggested that de novo PD patients present with reduced functional connectivity in putamen, indicating that drooling is a symptom of a widespread pathology [38], which is challenging to treat [2]. Future research should further examine the relationship between drooling and other aspects of the PD symptomatology [39,40], as well as the influence of other treatments commonly used in PD and to analyze their consequences on drooling [41].

## 2. Pathophysiology of Drooling

The processes of salivation are controlled by both sympathetic and parasympathetic nervous systems [42]. The process of salivary gland secretion involves primarily cholinergic signaling by the parasympathetic nerves and signaling by neuropeptides, such as substance P, but also adrenergic signaling by sympathetic nerves. Parasympathetic stimulation will activate acetylcholine receptors, and sympathetic stimulation will increase alpha-receptor stimulation, which causes smooth muscle contraction and increases volume flow [42].

Drooling is more pronounced in periods where patients are “off” medication [2]. The abnormality of salivary production and maintenance inside the oral cavity and inadequate salivary clearance are considered the two main major domains that impact the pathophysiology of drooling [2]. Saliva’s overproduction, by definition, can result in drooling. However, it has been found that PD patients produce less saliva compared to healthy controls [3,4,5], and the dopamine deficiency might explain why. However, the exact mechanism causing reduced salivary production has not been fully elucidated [4]. Studies using animal models have shown that saliva secretion is modulated by dopamine [6,7]. Specifically, the results of studies performed in rats have suggested that salivary secretion is a result of the activation of central and peripheral receptors of dopamine [7]. Lesion studies also support this claim, as a significant decrease in salivary secretion was identified when lesions in globus pallidus or its output pathway (lateral mesencephalic reticular formation) and the striatum were identified [8]. Aligned to this, a pathological study identified Lewy bodies in the superior cervical ganglion, the cervical sympathetic trunk, the peripheral vagus nerve, and the submandibular glands [9]. Another study by Costa et al. (2008) measured and compared the salivary production activity and velocity of salivary excretion of the parotic gland in healthy controls and PD patients, reporting the same production of saliva in both groups, but the parotic salivary excretion velocity to a distinct stimulus in the PD patients’ group was significantly higher when compared to the group of the healthy controls [43]. Therefore, the increased velocity of saliva excretion should not be the main contributor of drooling in patients with PD, but it might partially contribute to its pathophysiology.

The other major domain that can contribute to drooling is swallowing dysfunction during the oral and/or pharyngeal phase of swallowing. In PD patients, bradykinesia can lead to oropharyngeal dysphagia. A study in animal models found a slower tongue protrusion in the rat group injected with 6-hydroxydopamine (6-OHDA) when compared to healthy controls [10]. In addition, the parkinsonian rat group in another 6-OHDA videofluorographic study had higher rates of aberrant food bolus movement compared to healthy controls [11]. Furthermore, a study using videofluoroscopy (VFSS) suggested that the severity of dysphagia in drooling PD patients is directly correlated with drooling [12]. Therefore, impairment in the oropharyngeal phage of swallowing might be a main contributor to drooling’s pathophysiology. Furthermore, Kikuta T. et al. (2011) suggested that advanced PD patients present with lower maximum tongue pressure when compared to PD patients in early or moderate stages of their disease and that there is a negative correlation between the oropharyngeal transit time and the tongue movement [13]. Therefore, poor muscle control of the tongue and bradykinesia can contribute to the dysphagia’s pathophysiology and possibly to drooling itself. Hypomimia, involuntary mouth opening, stooped posture of the upper body, and dropped head can affect the patients’ ability to maintain saliva inside their oral cavity, and, therefore, cause drooling in PD [14]. Finally, studies using manometry suggested that impaired upper oesophageal sphincter (UES) mobility might also have an effect on dysphagia and drooling in PD patients. However, this cannot be the sole cause of dysphagia in patients who have adequate clearance mechanisms and pharyngeal propelling forces [15,16].

## 3. PD-Associated Symptomatology and Drooling

Drooling in PD patients correlates with other general clinical features, non-motor, and motor symptoms.

### 3.1. General Clinical Features

The prevalence of drooling ranges between 9.26% and 70% due to the disease heterogeneity and the different instrument measures used in every study [17,18,19,20,21,22,23,24,25,44,45,46]. It can manifest very early during the disease [20], but it is not considered a prodromal symptom in Parkinson’s according to the current MDS research criteria. Based on Braak’s staging of brain pathology in PD and the model of the hypothesized spread of alpha-synuclein (aSyn) in PD, aSyn accumulation begins in the gut and then progresses up to the brain via the vagus nerve [47]. Therefore, it can be suggested that gastrointestinal tract features should be a prominent early manifestation of PD. However, more scientific studies are required to better investigate whether drooling can contribute to the diagnosis of Parkinson’s.

The prevalence of drooling is higher in males [19,22,23,24] than females since women with PD showed a less malignant phenotype [48], with the estrogen activity in females probably delaying the development of the PD symptoms [49].

Moreover, the longer the disease duration [17,18,24,25] and progression, the higher the risk of drooling [14,17,18,22,24,25,26]. As drooling is mainly a result of a decreased frequency of saliva clearance inside the oral cavity, problems with posture [5], and oral motors, as well as facial impairment [31] (e.g., bradykinesia, rigidity, hypomimia), it is considered that all these deficits are more common and severe as the disease progresses.

Age is another crucial factor when it comes to prevalence. Drooling becomes more prevalent with age [17,22,24,25]. Any age-related changes can impact saliva control as we grow older. With aging, a natural progressive brain tissue loss that links up with neurological skills worsening and muscle-mass reduction occurs [50]. Therefore, the decreased muscle strength of the orofacial muscles (e.g., buccinator, tongue, and orbicularis oris) [50] can result in the accumulation of saliva inside the oral cavity and increase the possibility of anterior and posterior spillage of saliva. However, given that these studies did not include a control group, more scientific studies are required for more reliable results.

The prevalence of drooling is higher in PD patients with higher severity of Levodopa-induced dyskinesia (LID) [22,23]. This is shown in more advanced patients, where generally higher levodopa doses are used [14,17,18,22,24,25,26,27,51,52].

### 3.2. Non-Motor, Motor Symptoms, and Drooling

It has been suggested that a variety of non-motor and motor symptoms can occur during the course of Parkinson’s disease [53].

In regard to cognitive function, there have been inconsistent findings around the role of cognitive performance in drooling [17,22,23,25,28,29]; however, some studies suggested that drooling is linked to cognitive decline [27,30]. Specifically, Reynold et al. (2018) suggested that cognition has a role beyond the automatic process of drooling and saliva control [28]. They showed that divided attention impairment aggravates drooling in PD patients, using a paradigm where the vigilance of saliva control and frequency of saliva swallowing decreased during a distracting cognitive task [28]. Nevertheless, more studies are required to better understand if a closer link between cognition and drooling exists.

Sleeping disorders are associated with the appearance of drooling [22,23]. Good sleep quality in patients with PD was correlated with less motor symptoms in the morning [54]. Therefore, poor sleeping patterns can influence motors symptoms and promote drooling [22].

Dysautonomias, namely urinary disorders, sexual dysfunction [23], obstipation [22], other gastrointestinal manifestations, and orthostatic hypotension, were found to relate to drooling. The autonomic system is affected due to the alterations of the vagus nerve [31] and, therefore, it can result in several concomitant dysfunctions, such as drooling, gastrointestinal problems, and obstipation.

Speech difficulties [23] and dysphagia [17,23,25] are associated with drooling. Speech, swallowing, and saliva control share many organs and anatomical structures. Thus, impairment in one of them can result in dysfunction in all these areas. Muscles inside the oral cavity, as well as lips, tongue, jaw, cheeks, larynx, and pharynx, are impacted by rigidity, bradykinesia, and hypokinesia, usually present in PD [32,33]. A delayed swallowing reflux [34], lingual tremor, lingual pumping, prolonged lingual elevation, and mandibular excursion are observed changes in patients with PD and can contribute to reduced saliva control [35].

Hypomimia was also associated with drooling [24,26], and, therefore, lip closure reduction is noted in some of the PD patients affecting saliva control [36].

Bradykinesia was found to be linked to drooling [26], as it can impact the orofacial muscles [33]. As a result, the reduction of movements velocity of the lips, tongue, jaw, and cheeks, among others, can affect the control of saliva inside the oral cavity and its transport from the oral cavity to the oropharynx.

Patients with a PD dominant tremor did not present with a higher prevalence of drooling [22]. Nevertheless, according to one study, patients with PD non-dominant tremor presented with a higher prevalence of drooling [22]. These results can be attributed to the fact that non-dominant tremor patients exhibit a higher decrease in grey matter and neural functional connectivity related to motor regions [55], as well as an extensive Lewy bodies pathology in the cortical areas [56]. They also present with greater lingual control dysfunction and increased rigidity in the oropharynx [13].

Drooling was related to a more symmetric pattern PD presentation [24]. Patients with a higher burden of motor symptoms present with a more symmetric pattern [37], and, therefore, we can anticipate that drooling will also be more prevalent in such patients.

Interestingly, de novo PD patients with drooling have not been extensively investigated, and, therefore, drug treatment (e.g., Levodopa) might have affected the results. Levodopa is the main drug used in PD treatment; however, its long-term use can lead to dyskinesia and motor fluctuations [57]. Dyskinesia is generally progressive and can impact different areas of the body, including the orofacial muscles, the neck, the tongue, and the jaw [58]. Motor deficits resulting from dyskinesia in the aforementioned areas can enhance drooling.

In addition, the relation of DAT binding in the striatum has not been extensively investigated. Tajima et al. (2020) suggested that the severity of motor symptoms, especially axial symptoms (components of akinetic-rigid PD) and bradykinesia, but no tremor and specific binding ratio (SBR), in de novo PD may relate to drooling [59]. Therefore, it can be hypothesized that the mechanism of the drooling aggravation is similar to that of bradykinesia and axial symptoms since previous studies have shown that DAT binding correlates with bradykinesia and axial symptoms, but not with parkinsonian tremor [38,60,61]. However, the effects of Levodopa remain unclear [2,39], indicating that other mechanisms, in addition to the nigrostriatal dopamine system, play a role in drooling. An fMRI study (Hou et al., 2016) examined the functional connectivity in the basal ganglia of de novo PD patients with and without drooling. The drooling patients had significantly decreased functional connectivity in the putamen within sensorimotor cortices (bilaterally), the parietal (inferior and superior) lobules, and other areas in the occipital (right) and temporal (right) lobes [40]. Therefore, it can be inferred that drooling is a symptom of a widespread pathology, and it cannot be attributed to a single causing factor. As a result, the management of drooling is complex, as identifying treatment options to target such a widespread pathology can be challenging.

There have been suggested pharmacological and non-pharmacological treatments to tackle drooling in PD [2]. First, PD patients should withdraw cholinesterase inhibitors, namely quetiapine and clozapine, because they aggravate drooling [2]. Next, they should attempt to improve their motor symptoms (e.g., by using dopaminergic medication or performing deep brain stimulation (DBS)) [2]; nevertheless, it is important to highlight that there is no study that has specifically investigated the effect of DBS on PD patients with drooling. Behavioral modification and radiotherapy might also be used as complementary therapies [2]. However, all these treatments only partially contribute to the management of drooling and more specific treatment options are still required.

## 4. Limitations and Future Directions

It is eminent that future studies will use drooling-specific rating scales (e.g., Drooling Severity and Frequency Scale (DSFS), Sialorrhea Clinical Scale for PD (SCS-PD), and Drooling Rating Scale (DRS)) [2] as opposed to subjective data and patients’ complaints to evaluate drooling in PD. They should also include the examination of biochemical properties of saliva, its appearance, viscosity, flow, and volume, as well as the association between the degree of drooling severity and patients’ clinical characteristics. Interestingly, a study found that the Radboud Oral Motor inventory for Parkinson’s disease—Saliva (ROMP-saliva) scale is the only scale with data on patients with PD and clinimetric properties adequacy [62].

Moreover, other important aspects need further research. It would be interesting to examine the association between saliva production and smell, given that saliva secretion rate can be affected by smell [41] since the smell of food usually increases saliva production [63], and hyposmia is commonly observed in patients with PD. In addition to this, the association drooling has with fatigue and sensory deficits (i.e., vision abnormalities), as PD patients usually complain about those during the course of their disease [53]. It is essential to better understand whether drooling affects early PD patients so that we assess whether a correlation with a worse PD phenotype at the later disease stages occurs. Furthermore, more specific guidance is required around the pharmacological treatment of drooling and how the administration of botulinum toxin as the standard pharmacological treatment in drooling can have positive or negative impacts on other clinical features. Thus far, it is known that anticholinergic drugs to reduce drooling can have side effects, such as hallucinations or delirium [64]. Finally, including a control group would allow for more reliable results and safer conclusions.

## 5. Conclusions

The exact pathophysiology of drooling in patients with PD has not yet been fully explicated. More knowledge regarding how drooling is associated with clinical characteristics will help us understand whether such factors worsen drooling or are just concomitant. Excessive drooling has been associated with greater burdens of non-motor symptoms and increased severity of motor fluctuations and bradykinesia. A decrease of DAT binding in the striatum at DaTSCAN imaging has also been shown. All in all, excessive drooling in patients with Parkinson’s cannot be attributed to a single factor, but to a mixture of factors, as part of a widespread pathology that is complex to treat.
